# The Advantages of Next-Generation Sequencing Molecular Classification in Endometrial Cancer Diagnosis

**DOI:** 10.3390/jcm12237236

**Published:** 2023-11-22

**Authors:** Daniela Rivera, Michele Paudice, Giulia Accorsi, Floriana Valentino, Marta Ingaliso, Ada Pianezzi, Paola Roggieri, Lucia Trevisan, Giulia Buzzatti, Serafina Mammoliti, Fabio Barra, Simone Ferrero, Gabriella Cirmena, Viviana Gismondi, Valerio Gaetano Vellone

**Affiliations:** 1 Hereditary Cancer Unit, IRCCS Ospedale Policlinico San Martino, Largo Rosanna Benzi 10, 16132 Genova, Italy; mngiulia@hotmail.it (G.A.); flovalentino@alice.it (F.V.); ada.pianezzi@hsanmartino.it (A.P.); paola.roggieri@hsanmartino.it (P.R.); lucia.trevisan@hsanmartino.it (L.T.); giulia.buzzatti@hsanmartino.it (G.B.); gabriella.cirmena@hsanmartino.it (G.C.); viviana.gismondi@hsanmartino.it (V.G.); 2 Department of Surgical Sciences and Integrated Diagnostics (DISC), University of Genoa, Viale Benedetto XV 6, 16132 Genova, Italy; michele.paudice@unige.it (M.P.); s3511158@unige.it (M.I.); valerio.vellone@unige.it (V.G.V.); 3 Anatomic Pathology University Unit, IRCCS Ospedale Policlinico San Martino, Largo Rosanna Benzi 10, 16132 Genova, Italy; 4 Medical Oncology Unit 1, IRCCS Ospedale Policlinico San Martino, Largo Rosanna Benzi 10, 16132 Genova, Italy; serafina.mammoliti@hsanmartino.it; 5 Unit of Obstetrics and Gynecology, P.O. “Ospedale del Tigullio”—ASL4, Via G. B. Ghio 9, Metropolitan Area of Genoa, 16043 Chiavari, Italy; fabio.barra@icloud.com; 6 Department of Health Sciences (DISSAL), University of Genoa, Via Antonio Pastore 1, 16132 Genova, Italy; 7 Department of Neurosciences, Rehabilitation, Ophthalmology, Genetics, Maternal and Child Health (DiNOGMI), University of Genoa, Largo Paolo Daneo 3, 16132 Genova, Italy; simone.ferrero@unige.it; 8 Academic Unit of Obstetrics and Gynecology, IRCCS Ospedale Policlinico San Martino, Largo Rosanna Benzi 10, 16132 Genova, Italy; 9 Pathology Unit, IRCCS Istituto Giannina Gaslini, Via Gerolamo Gaslini 5, 16147 Genova, Italy

**Keywords:** endometrial cancer (EC), multigene-NGS panel, *POLE*, *TP53*

## Abstract

Endometrial cancer (EC) is the most frequent gynecological cancer. The ESGO/ESTRO/ESP 2020 guidelines identify prognostic groups based on morpho-molecular characteristics. This study aims to evaluate the clinical applicability of NGS analysis to define an appropriate risk class and to improve the diagnostic and prognostic stratification of ECs. Cases of serous carcinoma (OHEC) and high- (HGEC) and low-grade (LGEC) endometrioid carcinoma diagnosed with the morphological and immunohistochemical (IHC) protocols were considered. After a standardized pre-analytical phase, tumor DNA was semi-automatically extracted and analyzed using NGS with a panel of 14 genes. A total of 63 cases were considered. NGS analysis was successful in 60 cases; all of these were classified according to the new diagnostic algorithm. The molecular risk classification showed a good correlation with the morphological (k = 0.8). The study showed that the protocols of the pre-analytical and analytical phases used are robust and can lead to molecular results that fall within the standards required, which can be used in clinical practice for more precise diagnostic–therapeutic management of patients. The implementation of the classification is particularly relevant for better prognostic stratification of HGECs. In addition, the identification of a suspicious VUS in *POLE* questions the classification of truncating variants.

## 1. Introduction

Endometrial cancer (EC) is the most common gynecological cancer affecting women in developed countries. According to 2020 GLOBOCAN estimates, more than 417,000 new cases were diagnosed and nearly 97,000 women died worldwide from the disease. In Italy, EC is the third most common cancer in women aged 50–69 and about 10,200 new cases were estimated in 2022; it is expected to grow in the next few years to be the sixth most frequent cancer overall by 2030 [1,2,3]. Particularly, in the five major European markets (France, Germany, Italy, Spain and the United Kingdom), diagnosed EC incident cases are expected to increase from 42,775 cases in 2020 to 47,947 cases in 2030 at an annual growth rate (AGR) of 1.21% (Endometrial Cancer—Epidemiology Forecast to 2030, Published in May 2021; Report ID: 6073089).

Historically, EC was classified as a Type I or Type II carcinoma [4]. These two categories differ in several characteristics such as age of onset, risk factors, carcinogenesis, preneoplastic lesions, aggressiveness and prognosis. Type I encompasses estrogen-related tumors; these include low-grade endometrioid carcinomas (LGECs) and are usually diagnosed at an early stage with a good prognosis. They are characterized by several molecular alterations like *PTEN*, *KRAS*, *CTNNB1*, and *PIK3CA* pathogenic variations and microsatellite instability (MSI). Conversely, type II encompasses high-grade tumors usually diagnosed in an advanced stage and characterized by a worse prognosis. This group mainly consists of serous EC and is characterized by early *TP53* mutations [5]. However, this dualistic model has shortcomings in describing the complexity and heterogeneity of EC. In particular, it was noted that the high-grade endometrioid histotype showed intermediate immunomorphological features between the two groups [6]. 

EC diagnostic criteria have been determined by the WHO Classification System and are based on the morphological characteristics detected by microscopic diagnosis, which is easier and usually reproducible for LGECs, but given the considerable morphological variety present in HGEC tumors, diagnosis of the latter is harder. For these reasons, in 2013, The Cancer Genome Atlas (TCGA) proposed a new stratification based on genomic data, identifying four subgroups of EC with distinct genetic profiles: DNA polymerase ε (*POLE*, ultramutated), microsatellite instability (MSI, hypermutated), Copy Number Low and Copy Number High [7]. Even though the clinical outcomes are correlated to these molecular subgroups, the TCGA molecular classification is not feasible in routine diagnostic procedures. In order to create a more achievable diagnostic algorithm based on feasible techniques like immunohistochemistry (IHC) and Sanger or Next-Generation Sequencing (NGS) analyses [8,9,10], surrogate markers (*POLE* exonuclease domain mutation, loss of mismatch repair proteins expression, abnormal p53 expression and absence of the other markers) have been determined by other research groups. This led to the definition of four molecular prognostic groups: (i) the “*POLE*-mutated” (POLEmut) group, characterized by the most favorable prognosis and a good response to immune checkpoint inhibition; (ii) the “mismatch repair-deficient” (MMRd) group; (iii) the “no specific molecular profile” (NSMP) group; and (iv) the “*TP53*-mutant” (TP53mut) group, characterized by a poor prognosis [10,11,12,13].

As a result of this, the ESGO-ESTRO-ESP 2020 guidelines contain a revised stratification risk by introducing morpho-molecular data [14,15]. Even though the importance of molecular data has therefore been recognized, to date, only a few “hubs” can fully follow the recent guidelines to classify EC, mostly due to costs, time and limited experience related to molecular investigation of *POLE* in clinical practice. In particular, extensive sequencing, like whole-genome or whole-exome techniques, seems to be more suitable to address POLEmut genomic features of these carcinomas, but currently they are only rarely available in routine settings. *POLE* somatic mutations are present in 7–12% of ECs; these high-grade tumors are usually aggressive, but despite their morphological features, tend not to have aggressive behavior. POLEmut tumors have a typical mutational signature, mainly consisting of well-characterized hotspot mutations; most of them are missense substitutions, with a low proportion of small insertions/deletions [16].

In the last few years, several Next-Generation Sequencing (NGS) protocols have been developed to analyze tumor DNA. Multigene panel testing is a common approach to analyze cancer susceptibility genes, with good timing and a cost efficiency that are useful for EC molecular classification. In this scenario, we aimed to design and validate an NGS-multigene panel for EC; the most frequently mutated EC genes will be analyzed and the obtained results will be discussed in order to classify the lesions according to the revised TCGA classification criteria, defining an appropriate risk class. The combination of molecular data with established clinicopathologic risk factors could be useful for tailoring adjuvant therapy, especially in the high–intermediate risk group, for which clinical trials are currently under evaluation [17,18,19].

## 2. Materials and Methods

### 2.1. Study Cohort

Samples from all patients with a diagnosis of high-grade endometrioid EC (HGEC) and other high-grade endometrial carcinomas (OHECs) who underwent bilateral hysteron-adnexectomy in the period 2018–2020 were collected. In the latter group, only serous EC (the prototype of type II EC) samples were collected.

Then, the larger low-grade endometrioid EC (LGEC) cohort was consecutively chosen from 2018, matching the HGEC and OHEC cohort. All cases were reviewed by an expert in gynecological pathology (VGV and PM) and selected according to the following criteria: optimal fixation/storage, high representativeness of the entire neoplasia (higher than 30%), high tumor cellularity, low percentage of stroma cells, fibrosis and necrosis. Specimens were prepared according to standardized pre-analytical procedures [20]. Briefly, after surgical excision, all specimens were sent unfixed to the Pathology Unit, where they were fixed in 10% buffered formalin (12–18 h). After grossing, the samples were routinely processed and paraffin-embedded to obtain histologic slides stained in hematoxylin/eosin. The paraffin blocks were kept in dedicated archives at room temperature in cardboard boxes kept away from dust, light and heat sources. The most formalin-fixed paraffin-embedded (FFPE) blocks representative of the entire neoplasm were selected and manual macrodissection was performed.

### 2.2. DNA Extraction and NGS Sequencing

DNA was extracted from FFPE sections using automatic procedures (GeneRead DNA FFPE Treatment Kit on QIASymphony, Qiagen, Hilden, Germany). For samples with low amounts of starting material, a QIAamp DNA FFPE Tissue Kit was preferred. The DNA concentration was assessed via a Qubit 3.0 Fluorometer (ThermoFisher Scientific, Waltham, MA, USA) and the quality was assessed via an Agilent 4200 Tapestation with a high-sensitivity D1000 ScreenTape Kit (Agilent Technologies, Santa Clara, CA, USA). Samples with a concentration of <2.5 ng/µL along with a DNA Integrity Number (DIN) of <2 were excluded from sequencing. Molecular analysis was performed using NGS technology on an Ion Torrent S5 platform in combination with an Oncomine on-demand tumor-specific custom panel including 14 genes (*BRIP1*, *CTNNB1*, *KRAS*, *MLH1*, *MLH3*, *MSH2*, *MSH6*, *PALB2*, *PMS2*, *POLE*, *PTEN*, *TP53*, *RAD51C* and *RAD51D*). Genes were retrieved from the literature and selected on the basis of EC association [7,21,22]. Among these, a few genes associated with ovarian cancer (*BRIP1*, *PALB2*, *RAD51C*, *RAD51D*) were included in the panel for research purposes. Raw sequencing data were processed in Torrent Suite v.5.16.1 and variant annotation was performed in Ion Reporter v.5.20.0.

### 2.3. IHC Methods

IHC assays were performed on FFPE tissue sections using the automated ultraView Universal DAB procedure on the BenchMark ULTRA IHC/ISH Staining Module, Ventana. 

Two patterns of p53 expression (clone DO7, prediluted, Ventana, Cupertino, CA, USA) were considered: aberrant expression (diffuse strong nuclear positivity involving at least 80% of the tumor cells or complete absence of p53 expression with an internal positive control) and wild-type expression (variable proportion of tumor cell nuclei staining with a variable intensity). A p53 cytoplasmic pattern was not seen in any of these cases. MMR protein expression was evaluated with the following antibodies: MLH1 (Clone M1, Ventana), MSH2 (Clone G219, Ventana), MSH6 (Clone SP93, Ventana) and PMS2 (Clone A16-4, Ventana). A complete lack of tumor nuclear staining for one or more MMRPs (with internal positive control) was categorized as MMRP-deficient, while positive nuclear staining for all four MMRPs indicated an MMRP-retained status.

### 2.4. Variant Analysis and Classification

Parameters for analysis excluded variants with a variant allele frequency (VAF) < 5%, a coverage <500×, a quality score (PHRED) < 30, a strand bias > 0.65, a minor allele frequency (MAF) > 1% and a genomic position > 20 bp. Variants were classified as pathogenic/likely pathogenic (collectively termed pathogenic) according to the American College of Medical Genetics and Genomics (ACMG) recommendations [23] and Cancer Variant Interpretation Group UK (CanVIG-UK) Gene-Specific Guidance for *MMR* and *TP53* genes [24]. Variants of Uncertain Significance (VUSs) along with benign/likely benign variants were discarded. Copy number variations (CNVs) were not evaluated. All filtered variants were verified via visual inspection of bam alignment files in Alamut Visual Plus v.1.6. Variants with ambiguous allele frequencies were analyzed by Sanger sequencing and traces were visualized using MinorVariantFinder software (v.1.2.0-PRC-build-01) (ThermoFisher Scientific). 

### 2.5. Statistical Analysis

Associations of clinicopathological parameters with molecular subtypes were compared using a two-way Chi-squared test. For the concordance of EC risk profiles on a histo-morphological and molecular basis, the kappa value was calculated. The histopathological parameters of patients across the *TP53* mutation spectrum were compared using an Easy Fisher Exact Test Calculator. A *p* < 0.05 was considered statistically significant.

## 3. Results

### 3.1. Clinicopathological Characteristics

A total of 63 samples were selected for molecular analysis. The clinical and pathological characteristics of the 63 EC specimens are summarized in Table 1.

The median age was 72.1; considering age as a categorical variable related to the seniority threshold of 65 years, 43 patients (68.2%) were older and 20 (31.7%) were younger than 65.

**Figure 1:**
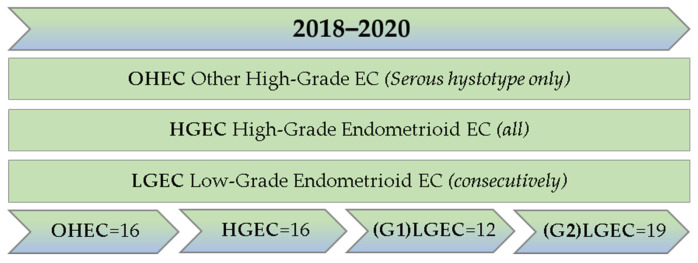
Case selection flow-chart.

Of the 63 cases, 47 (74.6%) were endometrioid and 16 (25.4%) were serous (Figure 1). The endometrioid cohort comprised 31 (65.9%) LGECs and 16 (34.1%) HGECs; among LGEC cases, 12 (38.7%) were G1, whereas 19 (61.3%) were G2. The OHEC cohort consisted of 16 cases, all of the high-grade serous histotype.

Regarding the stage of disease, 48 cases were stage I-II (76.2%) and 15 were stage III-IV (23.8%) according to the FIGO classification. Lymphovascular invasion (LVSI) was also evaluated, resulting in 23 (36.5%) positive and 40 (63.5%) negative cases. Among LVSI cases, 9 (39.1%) were classified as having “substantial” LVSI (involvement of >5 individual vascular spaces). 

The evaluation of MMR proteins highlighted 49 cases (77.8%) with preserved expression and 12 (19.0%) cases with MMRd, of which 11 had a loss of expression of MLH1/PMS2 (17.5%) and 1 (1.6%) had a loss of MSH2/MSH6. In two cases (3.2%), it was not possible to determine the status of the microsatellites. p53 expression was aberrant in 18 cases (28.6%), including 4 HGECs and 14 serous OHECs; the remaining 45 wild-type cases (71.4%) were LGECs (*n* = 31), HGECs (*n* = 12) and OHECs (*n* = 2).

### 3.2. Multigene-NGS Panel

All samples showed good quality parameters with DIN values ranging from 2.0 to 4.8 and DNA concentrations ranging from 2.66 to 81.2 ng/µL; hence, they were suitable for sequencing (Supplementary Table S1). Four separate sequencing runs were performed, with a mean depth of 2893 (range 1015–4980) and 93.5% target base coverage at 500×. Three samples were not compliant with the quality parameters, showing a mean depth of <1000 and a target base coverage at 500× of <60%, and were excluded from the subsequent analysis. Sequencing metrics along with the alignment quality are summarized in Supplementary Table S2; no technical replicates were performed. 

**Figure 2:**
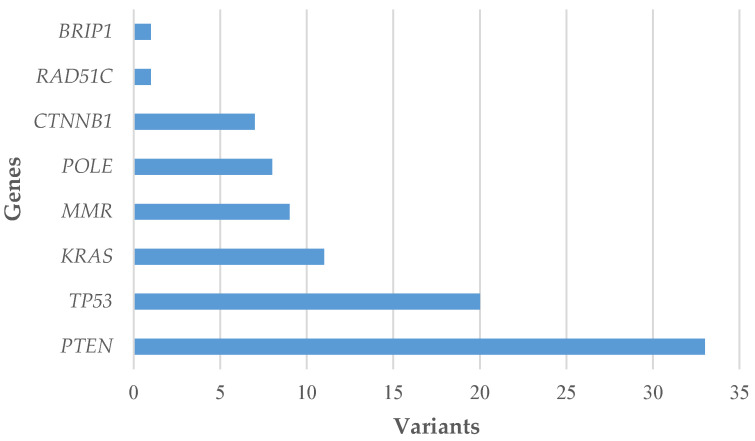
Pathogenic variants detected in EC cases.

The most frequently affected genes in our series were *PTEN* (55.0%) and *TP53* (33.3%), followed by *KRAS* (18.3%), *MMR* (15.0%), *POLE* (13.3%) and *CTNNB1* (11.7%); a pathogenic variant was also found in *RAD51C* (1.7%) and *BRIP1* (1.7%), both in association with other genes (Figure 2). All pathogenic variants are listed in Supplementary Table S3 and include 80 missense (67.8%), 35 (29.4%) stop-gained or frameshift and 3 (2.5%) splice-site variants. The Catalogue Of Somatic Mutations In Cancer (COSMIC) was interrogated to identify a correlation between recurrent variants (Supplementary Table S4) and histological classification, and no mutational signatures were identified [25].

### 3.3. Molecular Typing and Risk Classification

Based on the new integrated morpho-molecular classification [15], the three histogroups were categorized as POLEmut (8/60), MMRd (4/60), NSMP (30/60) and TP53mut (18/60). All cases were previously stratified for risk according to histopathological and morphological features; advanced metastatic and high–intermediate-, intermediate-, high- and low-risk classes were assigned (Supplementary Table S5). The molecular results allowed us to redefine the risk profile in eight cases (Supplementary Table S6); a statistically significant correlation (k = 0.818) was observed (Table 2). 

### 3.4. POLE and TP53 Profiles

In the 60 cases analyzed, 8 (15%) harbored a pathogenic variant in the exonuclease domain of *POLE* according to the literature [26,27,28,29]. The majority of these are endometrioid (four HGECs, three LGECs), while one (EC-49) was a serous OHEC. To the best of our knowledge, *POLE* pathogenic variants are very rarely described in ECs of serous histology [21], and some authors have supposed a possible diagnostic misclassification in case of *POLE* pathogenic variations [30]; therefore, a second histological evaluation was retrospectively performed, which revealed a morphologically “ambiguous” high-grade EC not fully framed as serous. A *POLE* pathogenic variant (c.857C > G; p.(Prp286Arg)) was also identified in an LGEC with an allele frequency below the threshold of 5%. In order to confirm or exclude this finding, a more accurate selection of the tumor area was performed; the Sanger sequencing traces were analyzed with Minor Variant Finder to determine variants as low as 5%, with negative results. 

Pathogenic variants in *TP53* were observed in 20 out of 60 (33.3%) cases, of which 6 were endometrioid (3 LGECs, 3 HGECs) and 14 were OHECs. Among the LGEC cases, two were carriers of additional clearly pathogenic variants, a *POLE* missense variant (EC-61) and two *MMR* variants (splice and nonsense variants, respectively; EC-33), so the risk profile was evaluated related to these findings. Notably, the serous OHEC cohort presented a mutation profile with *TP53* alone, whereas the endometrioid cohort presented *TP53* combined with other pathogenic variants (Table 3). There was a discordant case (EC-06) histologically classified as OHEC but carrying double pathogenic variants (*POLE* and *TP53*); a review of the IHC slides was required and a new HGEC phenotype was assigned. Consequently, the risk class also changed from “high” to “intermediate”. A statistically significant relationship was found between the *TP53* mutational status and immunohistochemical evaluations (*p* < 0.05), confirming the high value of p53 IHC as a molecular surrogate in clinical practice [31].

However, of the 20 cases harboring a mutation in *TP53*, two were not congruent with the IHC analysis (Supplementary Table S7). In particular, one case of serous carcinoma (EC-50) showed p53 expression in over 90% of the neoplastic elements (aberrant expression). The first NGS analysis resulted in the wild type, strongly disagreeing with the high reliability of the IHC results. For this reason, an area with a higher neoplastic density was selected in different sampling and DNA extraction, and a scrape from a glass slide was performed, identifying the c.796G > A; p.(Gly266Arg) pathogenic variant (VAF 18.25%). Conversely, in one case of LGEC (EC-58), the neoplasm showed a p53 expression of about 1%, and therefore it was considered “wild-type” by pathologists. However, the NGS analysis identified the c.734G > A; p.(Gly245Asp) pathogenic variant (VAF 29.15%), also confirmed by Sanger sequencing. This missense change is in the DNA-binding domain, and experimental studies have shown that it affects the *TP53* function [32,33,34,35,36,37,38,39]. This variant was reviewed by an expert panel (Accession VCV000012356.51) and classified as pathogenic. As already reported [40], IHC is therefore a reliable tool but is not always completely in agreement with molecular assays.

## 4. Discussion

The high heterogeneity of EC represents an important challenge in diagnostic settings and in the definition of the risk classification. Therefore, refs. [41,42] have highlighted the importance of a combined diagnosis on a morphological and molecular basis in order to precisely focus on the lesion for the definition of an appropriate risk class and to improve clinical management. Moreover, the FIGO 2023 staging system has recently been updated, integrating morpho-molecular features for endometrial staging.

Our study aimed to type DNA from FFPE samples via NGS sequencing in order to compare the molecular characteristics of the LGEC, HGEC and OHEC groups and to redefine the risk class of the patients. 

Our study population consists of 60 EC patients, the majority of which were LGEC patients (*n* = 31) followed by OHEC (*n* = 16) and HGEC (*n* = 13) patients, according to published guidance statements for the validation of NGS-based oncology panels [20,43]. 

The 14-gene NGS panel identified pathogenic variants in 57 out of 60 cases, with a mutation detection rate similar to the rates reported in the literature [21,30,44,45,46,47]. These results allowed us to classify our cohort with the new diagnostic algorithm for the integrated morpho-molecular classification of EC [15]. In particular, there were 20 (33.3%) tumors harboring a somatic pathogenic variant of *TP53* identified by NGS, of which 18 were classified as TP53mut. Despite the fact that several studies have shown a correlation between p53 IHC and the *TP53* mutation [48,49,50], we found an inconsistency in 2 out of 20 cases, highlighting how an appropriate molecular analysis carried out by personnel properly trained in oncological genetics is decisive in the diagnosis of a heterogeneous pathology such as EC. Regarding *MMR* status, our gene panel revealed four (6.7%) tumors with pathogenic variants in *MMR* genes, with only one concordant with the IHC results. Interestingly, all discordant cases (3/4) carried a truncating variant in *MSH6* with an allele frequency between 6 and 15%. This low allele frequency could justify the conservation of the protein in the tissue. The majority of our MMRd cases, in accordance with literature data [15], were characterized by the loss of *MLH1* and *PMS2* protein expression due to *MLH1* promoter methylation. For this reason, the inclusion of *MMR* genes in our NGS panel is not intended for detection of such alterations (no pathogenic variants found in these cases), but it may recover additional variations which would not be detected by immunohistochemistry. 

The POLE-mut tumors (8 out of 60, 15%) harbor pathogenic variants in the exonuclease domain of *POLE*, with allele frequencies ranging from 12% to 35%. All variants were reported in the literature [44] and designated as ‘hotspot’ *POLE* mutations. Castillo and colleagues reports them as frequently mutated in endometrial tissue, as already confirmed in COSMIC entries. Although they are reported as uncertain on dbSNP, it should be specified that in the somatic state, they could be considered as likely pathogenic. However, since there is a functional test that demonstrates a reduction in activity compared to the wild type, and also considering their localization, they can be classified as pathogenic [28]. We also identified a conspicuous fraction (28%) of VUSs in *POLE*; the majority are outside of the exonuclease domain. Nevertheless, there were two variants (c.901G > A; c.907C > T) in exon 9, both close to the splice junction and with a relatively low allele frequency (5.5% and 7.45%, respectively). The c.901G > A p.(Asp301Asn) variant was recorded but not classified in the ClinVar database (Variation ID: 405876) and is considered as a VUS in the ACMG Standards (https://varsome.com/) with one point applied to the PM2/PP3/BP1 supporting criteria. This alteration is predicted to be tolerated in in silico analyses, is not present in population databases and has not been reported in the literature in individuals affected with *POLE*-related conditions. Conversely, the c.907C > T p.(Gln303*) variant was reported as likely pathogenic in the Varsome database, but was recorded with conflicting interpretations of pathogenicity in ClinVar (Variation ID: 473841). This alteration is expected to result in a loss of function by premature protein truncation or nonsense-mediated mRNA decay. However, loss of function via haploinsufficiency in *POLE* has not yet been clearly established as a mechanism of disease. For these reasons, the clinical significance of *POLE*-truncating variants still remains unclear and functional studies to characterize their pathogenicity are needed. A higher percentage of cases were NSMP, with *PTEN* pathogenic variations significantly associated with endometrioid carcinomas, particularly LGEC. This finding strengthens the theory that *PTEN* mutations arise in an early stage of carcinogenesis of Type I carcinomas [47]. 

The NGS data confirmed the extreme heterogeneity and the different prognosis of high-grade endometrioid carcinomas [51]. In fact, HGECs were molecularly classified into three distinct classes: four POLEmut cases, six NSMP cases and three TP53mut cases. They can exhibit sluggish biological behavior if associated with a *POLE* pathogenic variant; on the contrary, if associated with a *TP53* pathogenic variant, they can be extremely aggressive, even overcoming serous carcinomas. Moreover, it is well known that some high-grade EC POLEmut may display morphological “serous-like” (i.e., EC-49) features and eventually “ambiguous” features between HGEC and serous OHEC. In this context, it is clear how the presence of POLEmut would crucially affect prognosis and patient management. On the other hand, serous OHECs have a confirmed molecular homogeneity, as they were almost all TP53mut, without any cases of MMRd, *PTEN* or *CTNNB1*. 

We collected clinical data for all eight POLEmut cases; as reported in Table 4, for all of these, no recurrences were reported (except the case with serous/ambiguous morphology), confirming the general favorable prognosis associated with POLEmut carcinoma. A good prognosis was also observed in an LGEC (EC-61), for which the indication for *POLE* genetic testing would be of minor importance considering the low-risk histotype and the stage of the disease. Of the remaining two EC cases with lower indication for testing, one was lost at the follow up; despite the low-grade malignancy, multi-infarct leukoencephalopathy was recorded for this patient and therefore the suspicion is of a poor prognosis.

The results we obtained allowed us to determine the risk classification on a molecular basis [15]. Comparison with the histological classification revealed that there is an excellent agreement (k = 0.818) between the two classifications. This applies to LGEC and OHEC, for which, in almost all cases, the risk class that had been assigned exclusively on a histo-morphological basis was confirmed. This points out how morphology is still important, and should be accompanied by a molecular analysis, which was confirmed to be crucial for the definition of the prognosis and follow-up [52,53,54,55]. The risk classification changed in five HGECs; among these, three were POLEmut and two were TP53mut. These data confirm again how HGECs are a highly heterogeneous group that need a molecular analysis in order to be stratified in the best possible way, since there are forms that fall within a group with a good or poor prognosis. 

There are some limitations of our study. The NGS panel we used does not cover some of the variations known to be associated with ECs, such as *ARID1A* [56]. In addition, we were unable to evaluate somatic copy number changes and the state of methylation of the *MLH1* promoter was not determined. Finally, the implementation of multi-omics data as well as functional assays to evaluate the pathogenicity of *POLE* variants might provide more useful subtyping. 

## 5. Conclusions

NGS technology for the identification of pathogenic variants in FFPE tissue gave good results and confirmed the feasibility of its clinical application.

The correct risk stratification, based on morphological, molecular and clinical parameters, associated with the early diagnosis of EC, allows for a better clinical–pathological management of the patient by better defining the risk class and relative treatment. This is especially true for high-grade endometrioid carcinomas, which comprise a heterogeneous group of neoplasms with markedly different prognoses. NGS analyses confirmed that HGECs are a heterogeneous group of neoplasms that exhibit intermediate morpho-molecular characteristics between LGECs and OHECs. Therefore, a molecular analysis of tumor tissue is particularly important to improve the diagnostic and prognostic definition of EC, especially if diagnosed in the early stage of the disease.

Furthermore, the molecular risk classification had excellent agreement (k = 0.818) with the histological classification for LGEC and OEHC. This confirms the importance of morphological data for a correct classification of lesions, which should be accompanied by a thorough molecular analysis, especially in genes associated with a favorable prognosis, such as *POLE*.

## Figures and Tables

**Table 1:** Clinicopathological features of the 63 EC cases analyzed in this study.

Characteristics	N	%
Age, years		
>65	43	68.2
<65	20	31.7
Histology		
Low-grade endometrioid EC (LGEC)	31	49.2
High-grade endometrioid EC (HGEC)	16	25.4
High-grade serous EC (OHEC)	16	25.4
FIGO stage		
I–II	48	76.2
III–IV	15	23.8
LVSI		
Negative	40	63.5
Positive	23	36.5
p53 expression		
Wild type	45	71.4
Aberrant	18	28.6
Microsatellite		
Conserved	49	77.8
Lost	12	19.0
Nd ^a^	2	3.2

^a^ Not determined.

**Table 2:** Concordance of molecular and histo-morphological evaluation of EC.

	Risk Profile (Molecular Class Unknown)					
Risk Profile (Molecular Class Known)	Advanced Metastatic	High	High–Intermediate	Intermediate	Low	*Total*
advanced metastatic	2	0	0	0	0	*2*
high	0	18	3	0	0	*21*
high–intermediate	0	0	4	0	0	*4*
intermediate	0	0	1	12	0	*13*
low	0	2	0	2	16	*20*
*Total*	*2*	*20*	*8*	*14*	*16*	60
Kappa: 0.81832						
Standard error: 0.05914						
95% CI: 0.70240 to 0.93424						

**Table 3:** Univariable associations of the *TP53* mutation profile with histological classification.

	*TP53* Alone	*TP53* Combined	*p*-Value
Serous	13	1	0.0022
Endometriod	1	5	

**Table 4:** Follow-up of the eight POLEmut cases.

Sample No.	Age (Years)	Follow-Up	Adjuvant Therapy	Staging	*POLE* Indication
EC-15-B	65	DWD ^a^	-	pT3a	No
EC-16-A	74	NED ^b^	-	pT1a/G3/N0(sn)	Yes
EC-36-A	60	NED	Radiotherapy	pT2/G1/N0(sn)	Yes
EC-38-A	73	LTFU ^c^	-	pT1a/G2/Nx	No
EC-42-A	54	NED	Radiotherapy	pT2G3pNx	Yes
EC-43-A	59	NED	Brachytherapy	pT1bG3pN0 LVSI+	Yes
EC-49-A	84	DWD	-	pT1bG3 “ambiguous”	Yes
EC-61-A	51	NED	-	pT1A/G2/N0(sn)	No

^a^ Died with disease; ^b^ no evidence of disease; ^c^ lost to follow-up.

## Data Availability

Data sets generated during this study as well as digital images of all cases stained with H&E are available from the corresponding author upon reasonable request.

## Supplementary Materials

The following supporting information can be downloaded at: https://www.mdpi.com/article/10.3390/jcm12237236/s1, Table S1—Quality parameters of the 63 EC specimen; Table S2—Sequencing metrics and alignment quality of the NGS runs; Table S3—List of pathogenic variants detected in EC cases; Table S4—Recurrent pathogenic variants identified in the 60 EC cases analyzed by NGS; Table S5—Risk profiles according to histopathological and morphological features of EC; Table S6—Risk profiles according to molecular classification of EC; Table S7—Concordance between TP53 molecular findings and IHC analysis.
