# Supplementary material for: The Advantages of Next-Generation Sequencing Molecular Classification in Endometrial Cancer Diagnosis
Source: J Clin Med. 2023 Nov 22;12(23):7236. doi: 10.3390/jcm12237236 (PMC10707080; doi:10.3390/jcm12237236)
Supplement: Supplementary file 1 [file jcm-12-07236-s001.zip › Supplementary Table S1.pdf]

Table S1 - Quality parameters of the 63 EC specimen

Sample no.	Histology no.	Tumor cell content [%]	DNA concentration [ng/µl]	DIN
EC-01-A	10027-2020/C4	70%	46.7	3.4
EC-02-A	6457-2020/B2	40%	9.45	2.4
EC-03-A	5609-2020/C9	70%	28.7	2.8
EC-04-A	15121-2019/B39	60%	8.95	2.0
EC-05-A	7754-2019/D1	80%	3.41	3.1
EC-06-A	7082-2019/A2	60%	2.66	2.1
EC-07-B	6524-2019/D23	90%	6.47	2.5
EC-08-A	261-2018/1-10	80%	50.0	2.9
EC-09-A	3388/18-A11.1	70%	17.7	2.0
EC-10-A	4425/18-A4.1	60%	5.21	2.5
EC-11-A	19204/18-A18.1	30%	47.5	4.8
EC-12-A	6001/19-11.1	45%	15.2	2.4
EC-13-B	6776/19-E3.1	70%	8.78	2.7
EC-14-A	15425/19-C2.1	70%	10.1	2.1
EC-15-B2	18824/19-C10.1	50%	6.43	2.7
EC-16-A	20497/19-C8.1	55%	39.4	4.8
EC-17-B	2087/18-1.9	60%	27.9	3.0
EC-18-B	10114/18-A12	80%	12.7	3.0
EC-19-B	19460/18-A10	50%	17.9	2.3
EC-20-A	20484/18-A21	70%	22.8	2.2
EC-21-A	26867/19-C28	70%	2.88	2.2
EC-22-B	5285/19-A17	50%	4.01	2.0
EC-23-B	6312/19-A6	35%	9.38	2.7
EC-24-B	13138/19-A11	35%	7.82	3.7
EC-25-A	15249/19-C5	60%	13.8	2.4
EC-26-A	18109/19-A11	60%	9.71	3.0
EC-27-A	24322/19-A7	50%	29.5	2.2
EC-28-B	24747-19/A7	60%	16.1	3.2
EC-30-B	6362/20-A7	70%	25.1	3.5
EC-31-A	17567/20-C9	70%	26.5	2.4
EC-32-A	11350/20-C2	65%	21.9	4.4
EC-33-A	1112/1-1.15	70%	67.2	2.2
EC-34-A	008848/18-A10	60%	43.9	2.1
EC-35-A	015945/18-A15	50%	56.0	2.1
EC-36-A	018894/19-C8	80%	55.2	2.2
EC-37-A	020159/19-A11	80%	60.0	2.2
EC-38-A	022633/18-A9	70%	5.08	2.4
EC-39-A	015848/19-A6	60%	23.3	3.2
EC-40-A	001858/19-A9	50%	31.3	2.4
EC-41-A	2020/011373 B8 1	70%	55.0	2.2
EC-42-A	2019/024820 A8 1	40%	54.0	2.4
EC-43-A	2020/006644 D1 1	60%	58.0	2.3
EC-44-A	2020/004135 A9 1	35%	53.0	2.5
EC-45-A	2020/006048 C21 1	50%	40.7	2.6
EC-49-A	2021-i00405	40%	49.2	2.5
EC-50-C	2021-I012659	65%	18.8	3.8
EC-51-A	2021-I016651	80%	44.3	2.7
EC-52-A	2021-I021925	60%	37.6	3.4
EC-53-A	PRF508270.01	70%	46.6	2.1
EC-54-A	2021-I021215	70%	46.5	3.2
EC-55-A	24452-17	80%	48.3	2.6
EC-56-A	29324-17	70%	23.5	2.4
EC-57-A	2019-I007330	50%	37.6	3.0
EC-58-A	2020-I001747	55%	35.4	2.8
EC-59-A	2018-I003824	60%	19.4	2.5
EC-60-A	2020-YR000002	70%	26.2	2.6
EC-61-A	2020-I016159	70%	34.8	2.4
EC-62-A	2020-I014923	50%	44.9	3.4
EC-63-A	2020-I020069	50%	7.45	2.9
EC-64-A	2020-I009004	60%	81.2	2.0
