# Supplementary material for: The Advantages of Next-Generation Sequencing Molecular Classification in Endometrial Cancer Diagnosis
Source: J Clin Med. 2023 Nov 22;12(23):7236. doi: 10.3390/jcm12237236 (PMC10707080; doi:10.3390/jcm12237236)
Supplement: Supplementary file 1 [file jcm-12-07236-s001.zip › Supplementary Table S2.pdf]

Table S2 - Sequencing metrics and alignment quality of the NGS runs

	run 1	run 2	run 3	run 4
total reads	24,188,008	24,335,061	22,236,676	22,705,820
polyclonal	26%	24%	29%	32%
final library	92%	92%	91%	95%
median read length bp	128	129	126	128
aligned bases	99%	99%	100%	99%
unaligned bases	1%	1%	0%	1%
mean raw accuracy 1X	99.6%	99.6%	99.6%	99.6%

	run 1			run 2			run 3			run 4		
	**AQ17**	**AQ20**	**Perfect**	**AQ17**	**AQ20**	**Perfect**	**AQ17**	**AQ20**	**Perfect**	**AQ17**	**AQ20**	**Perfect**
Total Number of Bases [bp]	2.79 G	2.69 G	2.41 G	2.85 G	2.75 G	2.47 G	2.57 G	2.47 G	2.22 G	2.67 G	2.54 G	2.22 G
Mean Length [bp]	121	119	110	123	121	112	119	117	109	122	119	108
Longest Alignment [bp]	355	354	340	370	370	344	358	358	322	361	361	339
Mean Coverage Depth [x]	0.9	0.9	0.8	0.9	0.9	0.8	0.8	0.8	0.7	0.9	0.8	0.7
