# Supplementary material for: The Advantages of Next-Generation Sequencing Molecular Classification in Endometrial Cancer Diagnosis
Source: J Clin Med. 2023 Nov 22;12(23):7236. doi: 10.3390/jcm12237236 (PMC10707080; doi:10.3390/jcm12237236)
Supplement: Supplementary file 1 [file jcm-12-07236-s001.zip › Supplementary Table S3.pdf]

Table S3 - List of pathogenic variants detected in EC cases

Sample no.	Genes	Coding	Amino Acid Change	Allele Frequency %	Exon	Coverage
EC-01-A	*TP53*	c.722C>T	p.(Ser241Phe)	67.95	7	2000
EC-02-A	*TP53*	c.659A>G	p.(Tyr220Cys)	75.4	6	2000
EC-03-A	*TP53*	c.817C>T	p.(Arg273Cys)	23.85	8	2000
EC-04-A	*TP53*	c.734G>T	p.(Gly245Val)	76.43	7	1345
EC-06-A	*TP53*	c.524G>A	p.(Arg175His)	50.35	5	2000
EC-07-B	*TP53*	c.839G>A	p.(Arg280Lys)	93.1	8	2000
EC-08-A	*TP53*	c.880G>T	p.(Glu294*)	94.64	8	1995
EC-09-A	*KRAS*	c.34G>T	p.(Gly12Cys)	49.88	2	800
	PTEN	c.388C>G	p.(Arg130Gly)	32.2	5	2000
	TP53	c.357del	p.(Lys120Serfs*3)	46.71	4	1989
EC-10-A	*PTEN*	c.101C>G	p.(Ala34Gly)	34.67	2	597
	PTEN	c.384G>C	p.(Lys128Asn)	41.62	5	1999
EC-11-A	*KRAS*	c.38G>A	p.(Gly13Asp)	10.49	2	1993
	PTEN	c.278A>G	p.(His93Arg)	24.59	4	732
EC-12-A	*KRAS*	c.35G>T	p.(Gly12Val)	37.42	2	1900
	PTEN	c.302T>C	p.(Ile101Thr)	22.59	5	580
EC-13-B	*TP53*	c.578A>G	p.(His193Arg)	80.85	6	1608
EC-14-A	*PTEN*	c.380_389delinsTAA	p.(Gly127Valfs*5)	57.51	5	1977
EC-15-B2	*POLE*	c.1231G>C	p.(Val411Leu)	12.95	13	1429
	MLH1	c.1103C>A	p.(Ser368*)	9.73	12	1141
EC-16-A	*MSH2*	c.832G>T	p.(Glu278*)	29.47	5	1758
	POLE	c.890C>T	p.(Ser297Phe)	25.95	9	1549
	PTEN	c.517C>T	p.(Arg173Cys)	26.6	6	2000
	PTEN	c.895G>T	p.(Glu299*)	25.04	8	1997
EC-17-B	*PTEN*	c.509G>T	p.(Ser170Ile)	25.61	6	1999
	PTEN	c.697C>T	p.(Arg233*)	13.7	7	2000
EC-18-B	*CTNNB1*	c.98C>G	p.(Ser33Cys)	13.56	3	1999
	PTEN	c.389G>A	p.(Arg130Gln)	12.96	5	1999
EC-19-B	*KRAS*	c.35G>A	p.(Gly12Asp)	8.54	2	773
	MSH6	c.1483C>T	p.(Arg495*)	6.35	4	788
EC-21-A	*PTEN*	c.37A>C	p.(Lys13Gln)	25.69	1	1997
	PTEN	c.365T>A	p.(Ile122Asn)	15.39	5	1995
EC-22-B	*PTEN*	c.389G>A	p.(Arg130Gln)	29.84	5	1535
	PTEN	c.955_958del	p.(Thr319*)	46.5	8	1757
EC-23-B	*PTEN*	c.202T>C	p.(Tyr68His)	18.58	3	1012
	PTEN	c.389G>C	p.(Arg130Pro)	17.55	5	2000
EC-24-B	*CTNNB1*	c.110C>G	p.(Ser37Cys)	7.65	3	2000
	PTEN	c.434_435insC	p.(Leu146Phefs*34)	23.43	5	1989
EC-25-A	*MSH6*	c.457+1G>A	p.(?)	8.34	2	1703
	MSH6	c.3132C>G	p.(Tyr1044*)	14.63	4	1032
EC-26-A	*PTEN*	c.377C>A	p.(Ala126Asp)	21.95	5	2000
EC-27-A	*PTEN*	c.388C>T	p.(Arg130*)	25	5	2000
	PTEN	c.517C>T	p.(Arg173Cys)	26.4	6	2000
	PTEN	c.741dup	p.(Pro248Thrfs*5)	27.62	7	1988
EC-28-B	*KRAS*	c.35G>T	p.(Gly12Val)	42.71	2	1997
	MSH2	c.1077A>T	p.(Arg359Ser)	81.46	7	1305
	MSH6	c.377C>A	p.(Ser126*)	25.8	2	2000
EC-30-B	*KRAS*	c.35G>A	p.(Gly12Asp)	16.32	2	1992
EC-31-A	*PTEN*	c.461del	p.(Phe154Serfs*5)	32.45	5	829
	PTEN	c.940G>T	p.(Glu314*)	30.65	8	2000
EC-32-A	*KRAS*	c.35G>T	p.(Gly12Val)	30.95	2	1554
	PTEN	c.388C>G	p.(Arg130Gly)	59.6	5	2000
EC-33-A	*MSH2*	c.2634+1G>T	p.(?)	6.87	IVS15	772
	MSH6	c.2731C>T	p.(Arg911*)	10.37	4	1379
	TP53	c.473G>A	p.(Arg158His)	6.39	5	1580
EC-34-A	*PMS2*	c.400C>T	p.(Arg134*)	6.77	5	1492
	PTEN	c.198G>T	p.(Lys66Asn)	5.25	3	2000
	PTEN	c.1038C>A	p.(Tyr346*)	5.06	9	1482
EC-35-A	*PTEN*	c.367C>G	p.(His123Asp)	39.1	5	2000
EC-36-A	*POLE*	c.1231G>T	p.(Val411Leu)	19.9	13	2000
	PTEN	c.389G>A	p.(Arg130Gln)	14.65	5	2000
	PTEN	c.414T>G	p.(Tyr138*)	9.65	5	2000
EC-37-A	*PTEN*	c.334C>G	p.(Leu112Val)	27.63	5	1987
	PTEN	c.388C>G	p.(Arg130Gly)	39.9	5	2000
EC-38-A	*CTNNB1*	c.109T>G	p.(Thr41Ile)	5.7	3	2000
	CTNNB1	c.122C>T	p.(Thr41Ile)	21.45	3	2000
	POLE	c.1231G>T	p.(Val411Leu)	35.15	13	2000
	PTEN	c.17A>C	p.(Lys6Thr)	39.85	1	5283

	PTEN	c.176C>A	p.(Ser59*)	30.20	3	1606
	PTEN	c.389G>A	p.(Arg130Gln)	36.3	5	2000
EC-39-A	*KRAS*	c.35G>T	p.(Gly12Val)	12.59	2	1104
	PTEN	c.955_958del	p.(Thr319*)	29.79	8	1974
EC-40-A	*KRAS*	c.35G>C	p.(Gly12Ala)	17.37	2	616
	PTEN	c.389G>A	p.(Arg130Gln)	30.1	5	2000
EC-41-A	*CTNNB1*	c.122C>T	p.(Thr41Ile)	37.11	3	1905
EC-42-A	*MSH6*	c.829G>T	p.(Glu277*)	17.50	4	2000
	MSH6	c.2836G>T	p.(Glu946*)	11.05	4	2000
	POLE	c.1331T>A	p.(Met444Lys)	29.83	13	1998
	PTEN	c.19G>T	p.(Glu7*)	30.62	1	1992
	PTEN	c.517C>T	p.(Arg173Cys)	12.56	6	1999
	PTEN	c.675T>G	p.(Tyr225*)	16.06	7	1999
	KRAS	c.520G>T	p.(Glu174*)	10.38	5	713
EC-43-A	*MSH2*	c.1738G>T	p.(Glu580*)	18.02	11	1998
	POLE	c.857C>G	p.(Pro286Arg)	17.45	9	2000
	PTEN	c.38A>C	p.(Lys13Thr)	7.53	1	3417
	PTEN	c.185A>C	p.(Lys62Thr)	13.76	3	1999
	PTEN	c.389G>A	p.(Arg130Gln)	18.81	5	1999
	PTEN	c.518G>A	p.(Arg173His)	9.4	6	2000
	RAD51C	c.364G>T	p.(Glu122*)	11.2	2	2000
EC-45-A	*PTEN*	c.697C>T	p.(Arg233*)	45.25	7	2000
	TP53	c.96+1G>C	p.(?)	34.66	3	1108
EC-49-A	*POLE*	c.1231G>T	p.(Val411Leu)	12.35	13	2000
	PTEN	c.389G>A	p.(Arg130Gln)	25.15	5	2000
EC-50-C	*TP53*	c.796G>A	p.(Gly266Arg)	18.25	8	2844
EC-51-A	*TP53*	c.524G>A	p.(Arg175His)	42.7	5	2000
EC-52-A	*TP53*	c.472C>G	p.(Arg158Gly)	78.05	5	2000
EC-53-A	*TP53*	c.844C>T	p.(Arg282Trp)	29.35	8	2000
EC-54-A	*TP53*	c.659A>G	p.(Tyr220Cys)	70.96	6	1632
EC-55-A	*TP53*	c.746G>T	p.(Arg249Met)	15.25	7	2000
EC-56-A	*KRAS*	c.40G>A	p.(Val14Ile)	5.64	2	780
	TP53	c.817C>T	p.(Arg273Cys)	55.15	8	2000
EC-57-A	*PTEN*	c.138C>G	p.(Tyr46*)	7.85	2	1999
	PTEN	c.388C>G	p.(Arg130Gly)	7.75	5	2000
EC-58-A	*PTEN*	c.287C>T	p.(Pro96Leu)	23.85	5	2000
	PTEN	c.389G>A	p.(Arg130Gln)	36.7	5	2000
	TP53	c.734G>A	p.(Gly245Asp)	29.15	7	2000
EC-59-A	*PTEN*	c.71A>G	p.(Asp24Gly)	15.86	1	1955
	PTEN	c.388C>G	p.(Arg130Gly)	21.63	5	1595
EC-60-A	*CTNNB1*	c.134C>T	p.(Ser45Phe)	16.14	3	1623
	PTEN	c.74_75insT	p.(Leu25Phefs*19)	11.62	1	1988
	PTEN	c.388C>T	p.(Arg130*)	16.8	5	2000
EC-61-A	*POLE*	c.1231G>C	p.(Val411Leu)	11.96	13	1998
	TP53	c.541C>T	p.(Arg181Cys)	9.04	5	1902
EC-62-A	*BRIP1*	c.1741C>T	p.(Arg581*)	52.38	12	1999
	CTNNB1	c.122C>T	p.(Thr41Ile)	9.5	3	2000
	PTEN	c.388C>G	p.(Arg130Gly)	24.1	5	2000
EC-63-A	*PTEN*	c.860C>G	p.(Ser287*)	7.5	8	2000
EC-64-A	*CTNNB1*	c.101G>A	p.(Gly34Glu)	11.5	3	2000
