# Supplementary material for: The Advantages of Next-Generation Sequencing Molecular Classification in Endometrial Cancer Diagnosis
Source: J Clin Med. 2023 Nov 22;12(23):7236. doi: 10.3390/jcm12237236 (PMC10707080; doi:10.3390/jcm12237236)
Supplement: Supplementary file 1 [file jcm-12-07236-s001.zip › Supplementary Table S4.pdf]

Table S4 - Recurrent pathogenic variants identified in the 60 EC cases analyzed by NGS

**Gene**	**Pathogenic variants**
CTNNB1	c.122C>T
KRAS	c.35G>A-C-T
POLE	c.857C>G
	c.1231G>C-T
PTEN	c.388C>G-T
	c.389G>A-C
	c.517C>T
	c.697C>T
	c.955_958del
TP53	c.524A>G
	c.659A>G
	c.817C>T
