# Supplementary material for: The Advantages of Next-Generation Sequencing Molecular Classification in Endometrial Cancer Diagnosis
Source: J Clin Med. 2023 Nov 22;12(23):7236. doi: 10.3390/jcm12237236 (PMC10707080; doi:10.3390/jcm12237236)
Supplement: Supplementary file 1 [file jcm-12-07236-s001.zip › Supplementary Table S5.pdf]

Table S5 - Risk profiles according to histopathological and morphological features of EC

**Histogroups**	**Risk profile (molecular class unknown)**					
	advanced-metastatic	*high*	*high-intermediate*	*intermediate*	*low*	*Total*
LGEC	0	1	4	10	16	*31*
HGEC	0	5	4	4	0	*13*
OHEC	2	14	0	0	0	*16*
Total	*2*	*20*	*8*	*14*	*16*	**60**

Chi-squared:	53,2851
Degrees of Freedom:	8
Significance level:	p< 0.0001
