# Supplementary material for: The Advantages of Next-Generation Sequencing Molecular Classification in Endometrial Cancer Diagnosis
Source: J Clin Med. 2023 Nov 22;12(23):7236. doi: 10.3390/jcm12237236 (PMC10707080; doi:10.3390/jcm12237236)
Supplement: Supplementary file 1 [file jcm-12-07236-s001.zip › Supplementary Table S6.pdf]

Table S6 - Risk profiles according to molecular classification of EC

Histogroups	Risk profile (molecular class known)				
	advanced-metastatic	*high*	*high-intermediate*	*intermediate*	*low*
LGEC	0	2	3	10	16
HGEC	0	7	1	2	3
OHEC	2	12	0	1	1
Total	*2*	*21*	*4*	*13*	*20*

Chi-squared:	33,4964
Degrees of Freedom:	8
Significance level:	p=0.0001

Total
31
13
16
**60**
