# Supplementary material for: The Advantages of Next-Generation Sequencing Molecular Classification in Endometrial Cancer Diagnosis
Source: J Clin Med. 2023 Nov 22;12(23):7236. doi: 10.3390/jcm12237236 (PMC10707080; doi:10.3390/jcm12237236)
Supplement: Supplementary file 1 [file jcm-12-07236-s001.zip › Supplementary Table S7.pdf]

Table S7 - Concordance between TP53 molecular findings and IHC analysis

	***TP53* molecular profile**	**IHC**
**EC-050**	Wild-type	Aberrant
**EC-058**	c.743G>A; p.(Gly245Asp)	Wild-type
